# Chemogenetic rectification of the inhibitory tone onto hippocampal neurons reverts autistic-like traits and normalizes local expression of estrogen receptors in the Ambra1+/- mouse model of female autism

**DOI:** 10.1038/s41398-023-02357-x

**Published:** 2023-02-20

**Authors:** Annabella Pignataro, Paraskevi Krashia, Margherita De Introna, Annalisa Nobili, Annamaria Sabetta, Francesca Stabile, Livia La Barbera, Sebastian Luca D’Addario, Rossella Ventura, Francesco Cecconi, Marcello D’Amelio, Martine Ammassari-Teule

**Affiliations:** 1 Institute of Translational Pharmacology, National Research Council, CNR, 00133 Rome, Italy; 2 IRCCS Santa Lucia Foundation, Centro Europeo di Ricerca sul Cervello CERC, 00143 Rome, Italy; 3 University Campus Bio-Medico, Rome, 00128 Italy; 4 Computational and Translational Neuroscience Laboratory, Institute of Cognitive Sciences and Technologies, National Research Council (CTNLab-ISTC-CNR), Rome, 00185 Italy; 5 Department of Psychology, University Sapienza, Rome, 00185 Italy; 6 Department of Biology, University of Rome ‘Tor Vergata’ 00133, Rome, Italy; 7 Cell Stress and Survival Group, Danish Cancer Society Research Center, DK-2100 Copenhagen, Denmark; 8 Department of Pediatric Hematology and Oncology, IRCSS Bambino Gesù Children’s Hospital, 00165 Rome, Italy; 9 Institute of Biochemistry and Cell Biology, CNR National Research Council, 00015 Rome, Italy

**Keywords:** Autism spectrum disorders, Neuroscience

## Abstract

Female, but not male, mice with haploinsufficiency for the proautophagic Ambra1 gene show an autistic-like phenotype associated with hippocampal circuits dysfunctions which include loss of parvalbuminergic interneurons (PV-IN), decrease in the inhibition/excitation ratio, and abundance of immature dendritic spines on CA1 pyramidal neurons. Given the paucity of data relating to female autism, we exploit the Ambra1^+/−^ female model to investigate whether rectifying the inhibitory input onto hippocampal principal neurons (PN) rescues their ASD-like phenotype at both the systems and circuits level. Moreover, being the autistic phenotype exclusively observed in the female mice, we control the effect of the mutation and treatment on hippocampal expression of estrogen receptors (ER). Here we show that excitatory DREADDs injected in PV_Cre Ambra1^+/−^ females augment the inhibitory input onto CA1 principal neurons (PN), rescue their social and attentional impairments, and normalize dendritic spine abnormalities and ER expression in the hippocampus. By providing the first evidence that hippocampal excitability jointly controls autistic-like traits and ER in a model of female autism, our findings identify an autophagy deficiency-related mechanism of hippocampal neural and hormonal dysregulation which opens novel perspectives for treatments specifically designed for autistic females.

## Introduction

Autism-spectrum disorders (ASD) are genetically heterogeneous pathologies of development that present common phenotypic characteristics including sensory and attentional deficits, behavioral rigidity, and global reduction of social interactions [1]. Gene linkage and proteomics studies show that a majority of genes identified as ASD risk factors are transcriptional, chromatin and synaptic genes [2] whose dysregulation accounts for the cytoarchitectural and synaptic abnormalities regularly observed in the brain of ASD patients and mouse models [3]. A central feature of ASD mice neural circuits is the disruption of the excitatory/inhibitory (E/I) balance [4, 5] due to a deficit in GABAergic inhibitory tone which triggers hyperexcitability in cortical [6] or cerebellar [7] principal neurons (PN). Remarkably, tuning the E/I balance to physiological levels via up- or downregulation of PV-IN or PN activity in mice with mutation in ASD-relevant genes rescues autistic behaviors [8–11].

We recently reported that haploinsufficiency of *Ambra1* (Ambra1^+/−^) gene, that encodes for activating molecule in Beclin1-regulated autophagy [12], results in sexual dimorphism of autistic traits with the ASD-like phenotype being restricted to the female mice [13]. At the behavioral level, Ambra1^+/−^ females show disruption of ultrasound communication, sociability defects, stereotyped behaviors, and defective reversal learning consistently with a reduction in behavioral flexibility [14, 15]. At the neural level, they exhibit hippocampal neuronal alterations which include a proliferation of immature dendritic spines and a reduction in the number of PV-IN that decreases the inhibitory control onto pyramidal PN and alters the E/I balance toward hyperexcitability [13].

Of note, confirming the construct-validity of this genetic mouse model for female autism, it has been reported that a single normal *AMBRA1* genotype, the intronic SNP rs3802890-AA, is associated with autism-related behaviors predominantly in women, who also display lower *AMBRA1* mRNA expression in blood [16].

Based on these evidences, we took advantage of Ambra1^+/−^ female mice to shed light on the pathogenic mechanisms specific to female autism. Specifically, we activated excitatory DREADDs in their residual pool of hippocampal CA1 PV-IN of PV_Cre Ambra1^+/−^ females to verify whether increasing the inhibitory control onto CA1-PN could rescue their neural and behavioral autistic-like traits. Then, considering that pathways involved in the female protective effect (FPE), like sex steroid female hormones [17], could be selectively altered in autophagy-deficient autistic females, we measured hippocampal expression levels of α and β estrogens receptors (ER) to examine their fluctuation upon variation of hippocampal networks activity.

## Materials and Methods

Procedure details for animals, genotyping, DREADDs (injections, and manipulations), electrophysiology, and dendritic spine analyses are provided in Supplementary Methods.

### Animals

**Fig. 1:**
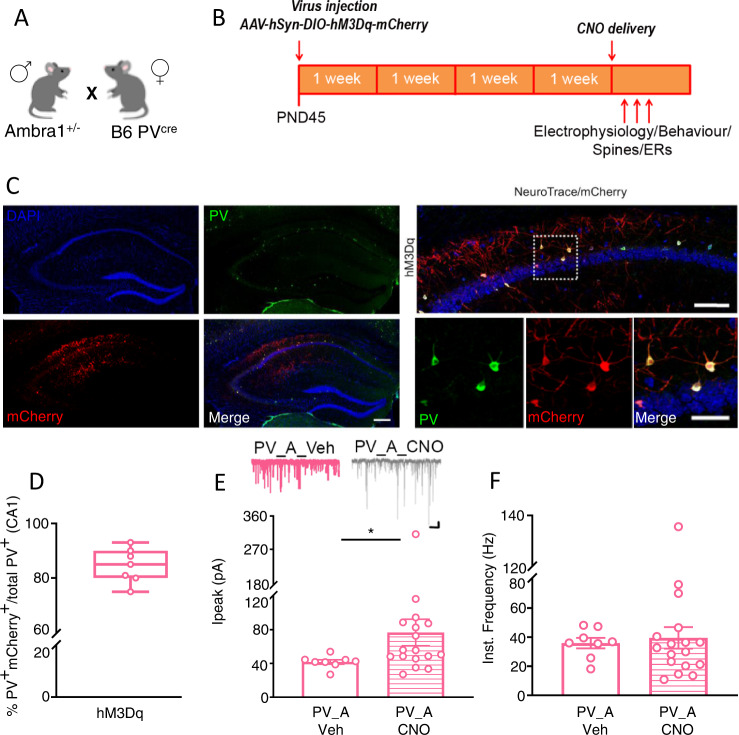
Chemogenetic activation of PV-IN enhances the GABAergic tone onto CA1 pyramidal neurons. **A** Breeding. **B** Experimental design: AAV-hSyn-DIO-hM3Dq-mCherry (hM3Dq) was bilaterally injected in CA1. Tests were performed 4 weeks later. Vehicle (Veh) or CNO were injected i.p. 40 min before behavioral testing and electrophysiological recordings, and 70 min before rtPCR experiments, or diluted at the same concentration in the drinking water 24 h before mice were sacrificed for dendritic spine analysis. **C** Left panels: low magnification image depicting the selective stereotaxic infusion of AAV-hSyn-DIO-hM3Dq-mCherry in the CA1. Scale bar: 200 μm. Right panels: representative images of CA1 NeuroTrace/PV/mCherry labelling showing overlapping signals (yellow) of mCherry (red) and PV + interneurons (green). Scale bars: top, 250 μm; below, 50 μm. **D** Percentage estimation of PV-IN infected with the hM3Dq AAV (PV^+^mCherry^+^/total PV^+^). In box-and-whisker plots the centre lines denote median values, edges are upper and lower quartiles, whiskers show minimum and maximum values and points are individual experiments (*N* = 7 for hM3Dq). **E**–**F** sIPSCs in CA1 neurons from hM3Dq PV_Ambra1^+/−^ females injected with Veh (left) or CNO (right). Scale bar: 1 s, 20 pA. CNO increased the inhibitory tone onto CA1 neurons. **E** Peak amplitude (*p* = 0.013, Mann-Whitney test). **F** Instantaneous frequency. *N* = 8, 17 neurons, from 3 Veh- and 5 from CNO females. Data are expressed as mean ± s.e.m. *p < 0.05.

Ambra1^+/−^ male mice were crossed with homozygous PV-Cre females (JAX stock #017320) to obtain PV-Cre mice expressing selectively Cre-recombinase in PV^+^ interneurons (Fig. 1A). Crossing and breeding procedures were compliant with the ethical guidelines of the European Council Directive (2010/63EU), the Italian Ministry of Health (Art.31, D. Lgs 26/2014) and ARRIVE guidelines. PV-Cre Ambra1^+/−^ mice and PV-Cre Wt mice were then referred to as PV_A and PV_Wt respectively. Individuals in each litter were first separated by sex and randomly assigned to each experimental condition until the chosen sample size was reached. All experimenters were blind to genotype and treatment during data collection and analyses.

### Genotyping

DNA was isolated from tail tissues and digested at 56 °C in the lysis buffer. DNA amplification and PCR products were analysed as in [13]. The presence of the PV-Cre construct was confirmed according to the Jackson Laboratory protocol (https://www.jax.org/).

### DREADDs manipulations

Manipulations of CA1 PV-IN activity were carried out in PV-Cre Ambra1^+/−^ (PV_A) and PV-Cre wild-type (PV_Wt) females by infusing sterotaxically in the CA1 region of the dorsal hippocampus [18] the adeno-associated virus (AAV) expressing the mutant human muscarinic receptor Gq with a fluorescent reporter (mCherry) in a Cre-recombinase-dependent manner (AAV-hSyn-DIO-hM3Dq-mCherry) due to the presence of a DIO cassette [19–21]. Manipulations of CA1 principal neurons activity were carried out in Ambra1^+/−^ and wild-type mice by infusing the inhibitory hM4Di receptor driven by the CamKII promoter (AAV5/CaMKIIa-hM4D(Gi)-mCherry, #AV6334 University of North Carolina Vector Core) in Ambra1^+/−^ females and Wt females. The injected volume in all manipulations was 0,7 μl.

#### CNO delivery

DREADDs were activated four weeks after viral vector injections by delivering the DREADDs ligand Clozapine N-oxide (CNO, 5 mg/kg dissolved in DMSO, C0832 Sigma-Aldrich) that was injected i.p. 40 min before behavioral testing and electrophysiological recordings and 70 min before rtPCR experiments or diluted at the same concentration in the drinking water 24 h before mice were sacrificed for dendritic spine analysis (Fig. 1B). PV_A and PV_Wt females infused with the AAV-hSyn-DIO-hM3Dq-mCherry and injected with, or drinking, the vehicle (Veh) instead of CNO were used as controls.

### Immunofluorescence and confocal microscopy

PV-IN or PN specificity of DREADDs activation was controlled by co-labelling of PV or αCamKII with mCherry in 30 μm-thick coronal sections as in [22]. Primary antibody: PV (1:500; Sigma-Aldrich; P3088); αCamKII (1:200; Thermo Fisher; #13-7300). Secondary antibodies: Alexa Fluor 488 donkey antimouse IgG (1:200; Thermo Fisher Scientific; #R37114), NeuroTrace 435/455 (1:200; Thermo Fisher Scientific; #N21479). Images were acquired by confocal laser scanning microscopy (Zeiss LSM 700; Carl Zeiss AG, Feldbach, Switzerland) and analyzed using ImageJ software (https://imagej.nih.gov/ij/).

### Electrophysiology

#### Bain slicing

Following halothane anesthesia, mice were decapitated and the brain was rapidly removed from the skull. Parasagittal brain slices containing the dorsal hippocampus (280 μm thickness) were obtained with a Leica VT1200S vibratome in chilled bubbled (95% O2, 5% O2) ice-cold sucrose-based solution (containing in mM): KCl 3, NaH2PO4 1.25, NaHCO3 26, MgSO4 10, CaCl2 0.5, glucose 25, sucrose 185; ~300 mOsm, pH 7.4).

#### Recordings in hM3D(Gq)-expressing PV-Cre_Ambra1^+/−^ females

A single brain slice was transferred to a recording chamber of an upright microscope (Axioskop 2-FS; Zeiss, Germany) and continuously perfused (3 mL sec-1, 32 °C) with aCSF. Whole-cell patch-clamp recordings were made from the soma of CA1 pyramidal neurons, identified using a magnification of 60x. All recordings were performed with Axon 700B amplifier using a 4 kHz low pass-filter, digitized at 20 kHz with a Digidata 1400 A and computer-saved using Clampex 10.3 (all from Molecular Devices, Sunnyvale, CA). No liquid junction potential correction was applied. Recording electrodes (3–4.5 MΩ) were pulled from thin-wall borosilicate glass tubes (TW150F-4; World Precision Instruments, Germany) and filled with (in mM): 140 CsCl, 1 MgCl2, 10 HEPES, 2.5 QX314-Cl, 4 Mg-ATP (~ 290 mOsm, pH 7.29). The extracellular solution for recording inhibitory currents was aCSF containing (in μM) 10 NBQX (Abcam), 50 D-AP5 (Abcam), 1 CGP55845 (Sigma-Aldrich) and 5 CNO to block the activity of AMPA/kainate, NMDA and GABAB receptors, respectively.

PV_A females were injected intraperitoneally with either vehicle (Veh: saline) or CNO 40 min before slicing. Spontaneous currents (sIPSCs) were recorded in voltage-clamp mode, holding the membrane potential at -70 mV. For analysis, one-minute-long analysis window was scanned for the detection of sIPSCs; single events were detected manually using an amplitude threshold crossing method in Clampfit 10.3 (Molecular Devices, Sunnyvale, CA) and analysed for amplitude, instantaneous frequency and charge transfer; all parameters were tested for time stability using Spearman’s rank order correlation test and segments of events that showed time instability during the experiment were excluded from further analysis. At least 400 events were analyzed for each experiment.

### Behavior

Social behavior was evaluated by exposing mice to the three-chamber (TC) and the social interaction in pairs (SIP) tests. Attention was evaluated in the Novel Object Recognition (NOR) test. Mice were tested during the diurnal cycle phase (10 am – 4 pm), 40 min after i.p. injection of CNO or Vehicle.

#### TC test

The apparatus consisted of three similar (70 cm L × 20 cm W × 20 cm H) adjacent chambers separated by two Plexiglas walls with small openings (5 cm W x 10 cm H) at the floor level allowing the mice to circulate between the chambers. The test included three phases of 10 min each (exploration of the apparatus, sociability, and social novelty) separated by 1 min intervals. In phase 1 (exploration) mice were placed in the central chamber and allowed to freely explore the empty apparatus. In phase 2 (sociability), one Plexiglas cylinder was placed in each external chamber; one of these cylinders was empty whereas the other one was containing a stranger wild-type C57BL/6 J female. In phase 3 (social novelty), the previously introduced female was left in the same cylinder and chamber whereas a novel C57BL/6 J female was placed in the formerly empty cylinder. The time spent sniffing the cylinder and the stranger mice (phase 2), and the familiar vs the novel female (phase 3) was manually recorded using chronometers. In phase 3, a recognition index (RI = time in contact with the unfamiliar female/time in contact with the unfamiliar + familiar female) was calculated [14, 23].

#### SIP test

The apparatus was a standard cage (26,5 cm L x 20,5 cm W, 14 cm H) where an Ambra1^+/−^ female was placed in isolation 5 days before the test [24]. The test consisted in introducing a stranger wild-type female (C57BL8/J) and measuring the total time (s) the experimental female interacted with the stranger female. Interactions included nose-to-nose contacts, and genital and body sniffing of the stranger female. The duration of the session was fixed at 5 min.

#### NOR test

The apparatus consisted of a squared Plexiglas cage (40 cm L × 40 cm W × 34 cm H). The test included three phases (phase 1: exploration of the empty apparatus; phase 2: training; phase 3: testing) of 5 min each separated by 1 min intervals. During training, mice were exposed to two objects identical in size, black/white colour pattern, and shape. During testing, mice were exposed to one previously explored (familiar) object left in its original location and a novel object of a comparable size but differing in black/white colour pattern and shape that was put at the location of the removed familiar object. Habituation of the open field exploration was estimated by measuring the velocity (cm/s) and the distance travelled using Noldus EthoVision XT software. Exploration of objects was measured by recording manually the time spent in contact with each identical object (training) and with the familiar and the novel object (testing) using chronometers. In phase 3 (testing), a recognition index (RI = time in contact with the unfamiliar object /time in contact with the unfamiliar + familiar object) was calculated.

### Dendritic spine analyses

Golgi staining was performed as in [13]. Dendritic spines were counted on randomly selected 30–50 μm dendritic segments of CA1 dendrites using the computer-based neuron tracing system (Neurolucida; MBF Bioscience, Williston, VT). Spine head diameters were measured on previously acquired images (Motic Live Imaging software) using ImageJ (NIH, USA) software. Spine head diameter values were expressed as cumulative frequencies. To avoid technical bias on spine head measurements, all groups were represented in a balanced manner in each staining experiment. To strengthen the validity of our observations, a subset of randomly selected dendritic spines for each group was analyzed using a recently reported new method [25] that allows efficient and unbiased classification of dendritic spines based on an objective basis that consider the unique geometry of different spine shapes. In more detail, we measured spine head width and length with the freely available RECONSTRUCT software (http://synapses.clm.utexas.edu.) [26]. After spine length and spine head width data sheet was constructed as in [26], we calculated the length-to-width ratio (LWR) to infer the spine category each spine was belonging to. The formula is hierarchical and classifies spines as follows: mushroom spine, when the width value >0.6 µm; long thin spine, when the length value >1 µm; thin spine, when the LWR value >1; stubby spine, when the LWR value ≤1.

### Hippocampal mRNA estrogen receptor levels

RNA purification and quantitative real time RT-PCR (qPCR) were used to measure hippocampal α and β estrogen receptor levels. In the DREADDs experiment, CNO injections were performed 70 min before sample collection, considering 40 min for DREADDs activation and further 30 min for RNA transcription. Total RNA was isolated from hippocampus brain punches using Total RNA purification Kit (Norgen Biotek, Thorold, Canada). RNA quantity was determined by absorbance at 260 nm using a NanoDrop UV–Vis spectrophotometer. For the reverse transcription of mRNAs (ER α/β and endogen control), random complementary DNA sequences were obtained using the High Capacity Reverse Transcription Kit (Applied Biosystems, Branchburg, NJ, USA). cDNA templates (8 ng for mRNA sample) were amplified by qPCR with the Taqman technology, using the 7900HT thermal cycler apparatus equipped with the SDS software version 2.3 (Applied Biosystems) for data collection. For ERα (Taqman assay: Mm00433149_m1) and for ERβ (Taqman assay:Mm00599821_m1) Ct values were normalized to averaged measures of Tata Binding Protein (TBP, Taqman assay ID Mm00446973_m1) [27]. All data were run in triplicate and were expressed as Fold Changes versus the control group, according to the ΔΔC(t) method [28].

### Statistics

All distributions were tested for normality by means of the Shapiro-Wilk normality test (GraphPad Prism). Parametric tests were used to compare data with normal distributions whereas non-parametric tests were used if the normality assumption was violated. Accordingly, recognition indexes (TC and NOR), time spent sniffing (SIP), spine density, percentage of thin spines, and levels of ER α and β measured upon DREADDs stimulation were compared between PV_A Veh, PV_A CNO, and PV_Wt Veh females by means of one-way ANOVAs, followed by the Bonferroni’s test for post-hoc pair comparisons. The percentage of mushroom, stubby and long-thin dendritic spines was compared between the same three groups by means of the Kruskal-Wallis test, followed by the Dunn’s test for post hoc pair comparisons. Mann–Whitney tests were used to compare sIPSC amplitude and frequency. Wilcoxon rank sum test or two-tailed Student’s t-tests for paired samples were used to compare within each group the exploration time of different items (raw data) in the social novelty and sociability phase of the TC, and in phases 2 and 3 of NOR. Comparisons of spine head diameters were carried out by means of the Kolmogorov-Smirnov (KS) test. Levels of ER α and β in basal condition were compared by means of two-tailed Student’s t-tests for independent samples. Data collection stopped when sample size was reached. These was determined in each experiment by setting the probability of a Type I error (α) and power at 0.05 and 0.80, respectively.

## Results

### Injection of the excitatory DREADDs in PV-Cre Ambra1^+/−^ females triggers a high rate PV-IN infection

The infection rate produced by the AAV-hSyn-DIO-hM3Dq-mCherry infusion was estimated by co-labelling of PV with mCherry. ImageJ processing revealed that 84,6% of PV-positive interneurons (green signal) were expressing the excitatory DREADD (mCherry red signal) (Fig. 1C and D). No off-target signal was detected in regions proximal to the injection site.

### Chemogenetic activation of PV-IN enhances the GABAergic tone onto CA1 pyramidal neurons in Ambra1^+/−^ females

To verify that chemogenetic activation of PV-IN enhanced the GABAergic tone onto CA1 principal (PN) neurons, we recorded spontaneous inhibitory postsynaptic currents (sIPSCs) from CA1 pyramidal neurons in PV_A female mice injected intraperitoneally with Veh or CNO. Consistently with a DREADD-induced potentiation of the GABAergic inhibitory input, sIPSCs amplitude was increased in CNO-injected PV_A females in comparison with PV_A females injected with the vehicle (Fig. 1E) whereas no increase in frequency was found (Fig. 1F).

### Chemogenetic activation of PV-IN rescues social behaviors in Ambra1^+/−^ females

We next sought to determine whether DREADDs enhancement of PV-IN activity could rescue impairments in social behavior previously reported in Ambra1^+/−^ females exposed to the three-chamber (TC) and the social interaction in pairs (SIP) tests [13].

#### TC

**Fig. 2:**
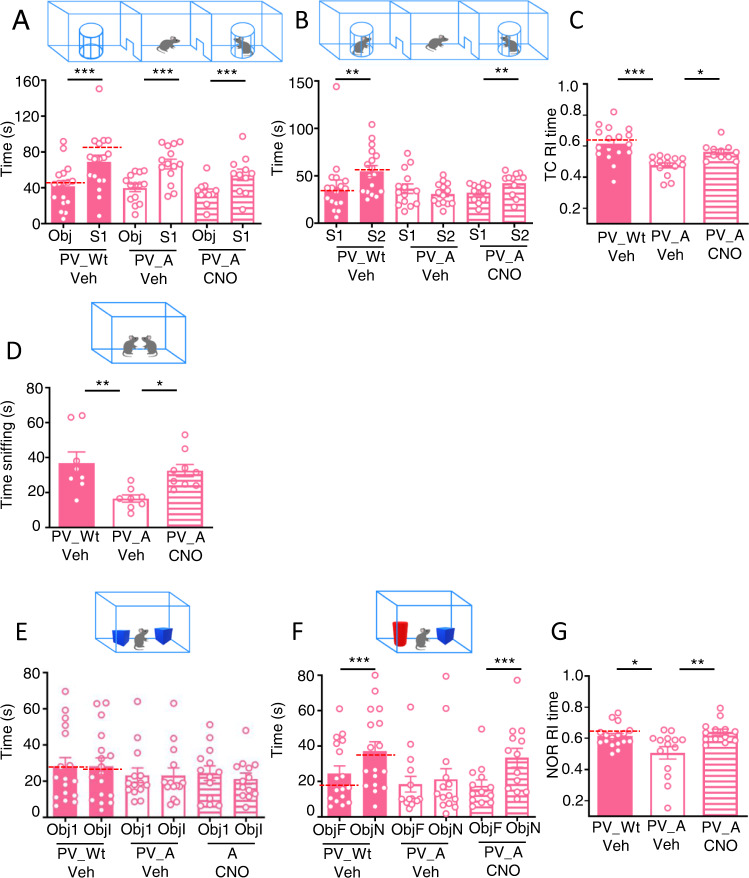
Chemogenetic activation of inhibitory PV-IN in Ambra1^+/−^ females normalizes autistic-like behaviors. **A** Histogram report mean time spent exploring the object (Obj) and the conspecific stranger (S1) during the sociability phase of the TC test. All mice explore more the conspecific stranger than the object (PV_Wt/Veh: Obj vs S1: t_(16)_=7.116, *p* = 0.0001; PV_A/Veh: Obj vs S1: t_(13)_=7.714, *p* = 0.0001; PV_A/CNO: Obj vs S1: t_(10)_=5.507, *p* = 0.0003; Dotted-line indicate mean values detected in PV_Wt/CNO control mice infused with vehicle instead of DREADDs. **B** Histogram report mean-time spent exploring the familiar female (S1) and the novel female (S2) during the social novelty phase of the TC test. Only PV_A/Veh females show impairment in recognition of the social novelty as they spent equal amounts of time sniffing S1 and S2. (PV_Wt/Veh: S1 vs S2: W = 119, *p* = 0.0032; PV_A/Veh: S1 vs S2: t_(13)_=1.740, *p* = 0.1; PV_A/CNO: S1 vs S2: W = 55, *p* = 0.002). Dotted-line indicate mean values detected in PV_Wt/CNO control mice infused with vehicle instead of DREADDs. **C** Histogram reporting the mean recognition index (RI) calculated during the social novelty phase of TC. Social novelty impairments in A/Veh females are rescued in A/CNO females. TC social novelty phase: PV_Wt/Veh (*N* = 17), PV_A/Veh, (*N* = 14), PV_A/CNO (*N* = 11), F_(2,39)_=11.26 *p* = 0.0001; PV_Wt/Veh vs PV_A/Veh, *p* = 0.00086; PV_A/Veh vs PV_A/CNO, *p* = 0.037. Dotted-line indicate mean values detected in PV_Wt/CNO control mice infused with vehicle instead of DREADDs. **D** Histogram reports mean time spent exploring an unknown conspecific during the social interaction in pair test (SIP). Social interaction impairments of PV_A/Veh females in SIP test are rescued in PV_A/CNO females (SIP: PV_Wt/Veh (*N* = 8), PV_A/Veh (*N* = 8), PV_A/CNO (*N* = 9), F_(2,22)_=6.186, *p* = 0.007; PV_Wt/Veh vs PV_A/Veh, *p* = 0.009; PV_A/Veh vs PV_A/CNO, *p* = 0.04). **E** Histogram report mean time spent exploring the two identical objects (Object 1 (Obj1) and Identical Object (Obj I)) during the object exploration phase of the NOR test by PV_Wt/Veh females and PV_A females injected with Veh or CNO. Data indicate that regardless of genotype and treatment all mice similarly explore the identical objects (PV_Wt/Veh: Obj1 vs ObjI: W = 7, *p* = 0.88; PV_A/Veh: Obj1 vs ObjI: W = -38, *p* = 0.24; PV_A/CNO: Obj1 vs ObjI: t_(12)_=1.102, *p* = 0.29). Dotted-line indicate mean values detected in PV_Wt/CNO control mice infused with vehicle instead of DREADDs. **F** Histogram report mean time spent exploring the familiar object (ObjF) and the novel object (Obj N) during the test phase of the NOR by PV_Wt/Veh females and PV_A females injected with Veh or CNO. Data indicate that recognition impairments of the novel object in A/Veh females are rescued in A/CNO females. (ObjF vs ObjN: PV_Wt/Veh: t_(16)_=7.06, *p* = 0.0001; PV_A/Veh: W = -29, *p* = 0.38; PV_A/CNO: W = -91, *p* = 0.0002). Dotted-line indicate mean values detected in PV_Wt/CNO control mice infused with vehicle instead of DREADDs. **G** Histogram reporting the mean recognition index (RI) calculated during the test phase of NOR test. Novel object recognition impairments in PV_A/Veh females are rescued in PV_A/CNO females. (PV_Wt/Veh (N = 17), PV_A/Veh (N = 14), PV_A/CNO (*N* = 13), F_(2,41)_=6.59, *p* = 0.003; PV_Wt/Veh vs PV_A/Veh, *p* = 0.023; PV_A/Veh vs PV_CNO, *p* = 0.004). Dotted-line indicate mean values detected in PV_Wt/CNO control mice infused with vehicle instead of DREADDs. Data are expressed as mean ± s.e.m. *p < 0.05, **p < 0.01, ***p < 0.001.

During the sociability phase all mice groups spent more time sniffing the stranger wild-type female than the object (Fig. 2A). Between-group differences were instead found during the social novelty phase where Veh-PV_Wt females interacted more with the novel female than the familiar one whereas Veh-PV_A females did not, as shown by the similar distribution of the time spent exploring the two females (Fig. 2B) and by the significant decrease of their recognition index (RI) (Fig. 2C). Remarkably, CNO-PV_A females behaved as did Veh-PV_Wt females, consistently with the rescuing effect of DREADD manipulation. To control for any side effect of CNO on mice behavior, we run an additional control group consisting of PV_Wt mice infused with veh (PBS 1x) in CA1, injected with CNO, and exposed to the task 40 min later. Remarkably, mice from this group behave as did PV_Wt mice infused with AAV-hSyn-DIO-hM3Dq-mCherry and injected with Veh during both the sociability and social novelty phases, thereby excluding any effect of CNO per se on behavior (Dotted-line in Fig. 2A–C histograms and Supplementary Figure S1A–C).

#### SIP

The SIP test consists in monitoring the time a PV_A female reared in isolation spends sniffing a stranger C57BL/6 female. As shown in Fig. 2D, Veh-PV_Wt females spent more time sniffing the stranger female than did Veh-PV_A females which sniffed less, and rapidly stopped any approach. As in the TC test, the SIP deficit was fully rescued in CNO injected PV_A females which behaved as did Veh-PV_Wt females.

### Chemogenetic activation of PV-IN rescues attention in Ambra1^+/−^ females

Attention deficit is a core symptom of ASD patients [1, 3] and male mice with deletion of ASD-relevant genes [29]. Although it has been reported that 30 week-old Ambra1^+/−^ females show intact performance in a NOR task where only one object is presented during training, and two objects, the familiar and a novel one, are presented during testing [14], we wondered whether a NOR deficit could be pinpointed in our younger Ambra1^+/−^ females using the standard NOR task given that, encoding and comparing the features of similar and different pairs of objects might be more effortful. We found that all groups show the same velocity and distance traveled during the habituation phase (Supplementary Figure S1D) and equally explored the identical objects during the training phase (Fig. 2E). Differently, during the test phase (Fig. 2F and G), Veh-PV_Wt females explored more the novel object than the familiar object whereas Veh-PV_A did not, as shown by the significant reduction in time spent sniffing the novel object (Fig. 2F) and by the significant decrease of their recognition index (RI) (Fig. 2G). As in TC and SIP tests, the NOR deficit was fully rescued in CNO-PV_A females which behaved as did Veh-PV_Wt females.

We also controlled for side effects of CNO in the NOR task and observed that, like in the TC task, PV_Wt mice infused with veh (PBS 1x), and injected with CNO performed as did PV_Wt mice infused with AAV-hSyn-DIO-hM3Dq-mCherry and injected with Veh in each NOR 2 and 3 (Fig. 2E–G, Dotted-lines and Supplementary Figure S1E–G).

### Chemogenetic decrease of CA1 principal neuron activity replicates the behavioral rescue produced by chemogenetic increase of PV-IN activity

**Fig. 3:**
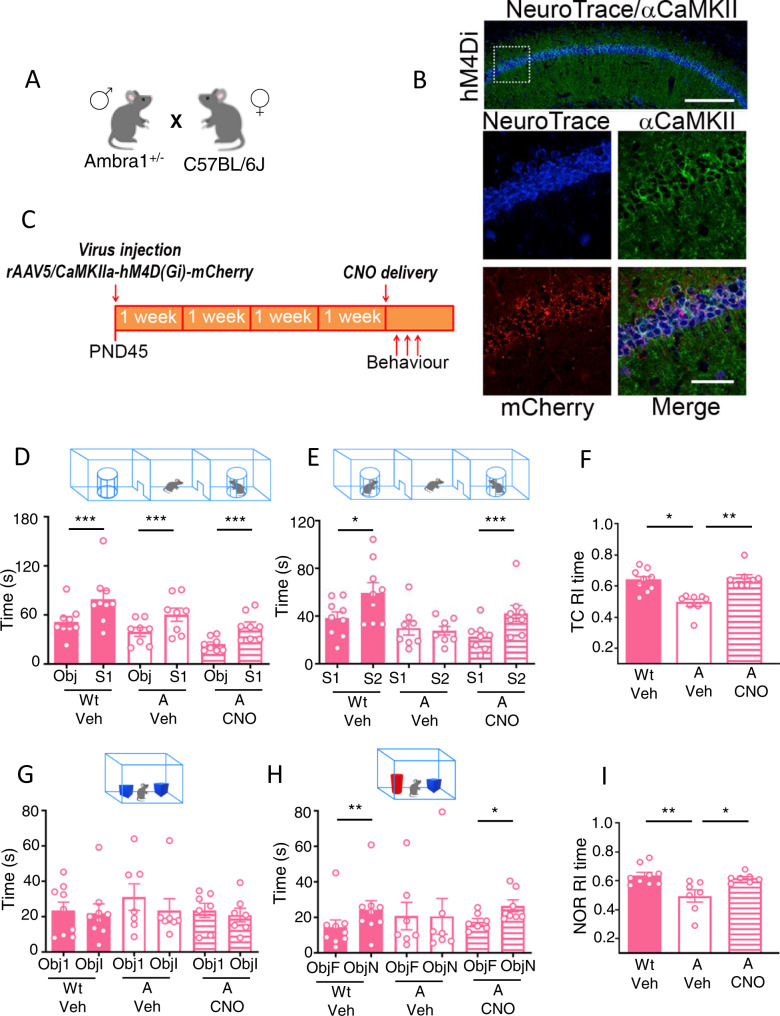
Chemogenetic inhibition of CA1 principal neuron activity in Ambra1^+/−^ females rescues autistic-like behaviors. **A** Breeding. **B** Representative images of NeuroTrace/CamKII/mCherry labelling showing overlapping signals (yellow) of mCherry (red) and CamKII- neurons (green). hM4Di receptors are selectively expressed in CA1 principal neurons. Scale bars: top, 200 μm; below, 50 μm. **C** Experimental design: the inhibitory rAAV5/CaMKIIa-hM4D(Gi)-mCherry vector was bilaterally injected in CA1 of Wt and Ambra1^+/−^ females. Behavioral tests were performed 4 weeks later. Vehicle (Veh) or CNO were injected i.p. 40 min before testing. **D** Histogram reporting mean time spent exploring the object (Obj) and the conspecific stranger (S1) during the sociability phase of the TC test. All mice explore more the conspecific stranger than the object (Wt/Veh: Obj vs S1: t_(8)_=5.43, *p* = 0.0006; A/Veh: Obj vs S1: t_(7)_=7.389, *p* = 0.0002; A/CNO: Obj vs S1: t_(7)_=5.531, *p* = 0.0009). **E** Histogram reports mean time spent exploring the familiar female (S1) and the novel female (S2) during the social novelty phase of the TC test. Only A/Veh females show impairment in recognition of the social novelty as they spent equal amount of time sniffing S1 and S2. (Wt/Veh: S1 vs S2: t_(8)_=2.517, *p* = 0.036; A/Veh: S1 vs S2: t_(7)_=0.562, *p* = 0.59; A/CNO: S1 vs S2: t_(7)_=5.251, *p* = 0.001). **F** Histogram reporting the mean recognition index (RI) calculated during the social novelty phase of TC. Social novelty impairments in A/Veh females are rescued in A/CNO females. (TC social novelty phase: Wt/Veh (*N* = 9), A/Veh (*N* = 8) and A/CNO (*N* = 8); Kruskal-Wallis H = 12.01, *p* = 0.002; Dunn’s multiple comparison Wt/Veh vs A/Veh, p = 0.029; A/Veh vs A/CNO, *p* = 0.005). **G** Histogram reports mean time spent exploring the two identical objects during the object exploration phase of the NOR test by Wt and A females injected with Veh or CNO. Data indicate that regardless of genotype and treatment all mice similarly explore the identical objects (Obj1 vs ObjI: Wt/Veh: W = -9, *p* = 0.63; A/Veh: W = -17, *p* = 0.17; A/CNO: t_(6)_=0.98, *p* = 0.36). **H** Histogram reports mean time spent exploring the familiar object (ObjF) and the novel object (ObjN) during the test phase of the NOR by Wt/Veh females and A females injected with Veh or CNO. Data indicate that recognition impairments of the novel object in A/Veh females are rescued in A/CNO females (ObjF vs ObjN: Wt/Veh: W = 45, *p* = 0.0039; A/Veh: W = 12, *p* = 0.37; A/CNO: W = -26, *p* = 0.031). **I** Histogram reporting the mean recognition index (RI) calculated during the test phase of NOR test. Novel object recognition impairments in A/Veh females are rescued in A/CNO females (Wt/Veh (*N* = 9), A/Veh (*N* = 7), and A/CNO (*N* = 7); F_(2,20)_=7.473, *p* = 0.0038; Wt/Veh vs A/Veh *p* = 0.004 A/Veh vs A/CNO, *p* = 0.021). Data are expressed as mean ± s.e.m. *p < 0.05, **p < 0.01, ***p < 0.001.

Neural network hyperactivity due to decreased inhibitory input onto PN is a complex process that does not solely depend on dysfunctional PV-IN which, in the CA1 region of the hippocampus, represent about 25% of the GABAergic interneuron population [30]. To ascertain that hippocampal PV-IN are main players in rescuing the autistic traits of Ambra1^+/−^ females, we verified whether direct/inverse chemogenetic manipulations of CA1 PN could replicate the rescue of social behavior and attention produced by increasing PV-IN activity. Accordingly, we infused the inhibitory hM4Di receptor driven by the CamKII promoter in the CA1 region of Ambra1^+/−^ females that were then injected with CNO or Veh, and in Wt females that were then injected with Veh (Fig. 3A–C).

In the TC test, all groups spent more time in contact with the stranger female than with the object during the sociability phase (Fig. 3D). Group differences were found in the social novelty phase where Veh_A females failed to show social novelty preference whereas CNO_A females succeeded and behaved as did Veh_Wt females (Fig. 3E and F). In the NOR task, all groups showed the same velocity and distance traveled during the habituation phase (Supplementary Figure S1H), and equally explored the similar objects during the training phase (Fig. 3G). Group differences were found in the test phase where Veh_A females failed to explore more the novel object than the familiar object whereas CNO_A succeeded and behaved as did Veh_Wt females (Fig. 3H and I).

### Chemogenetic activation of PV-IN rescues dendritic spines abnormalities in Ambra1^+/−^ females

**Fig. 4:**
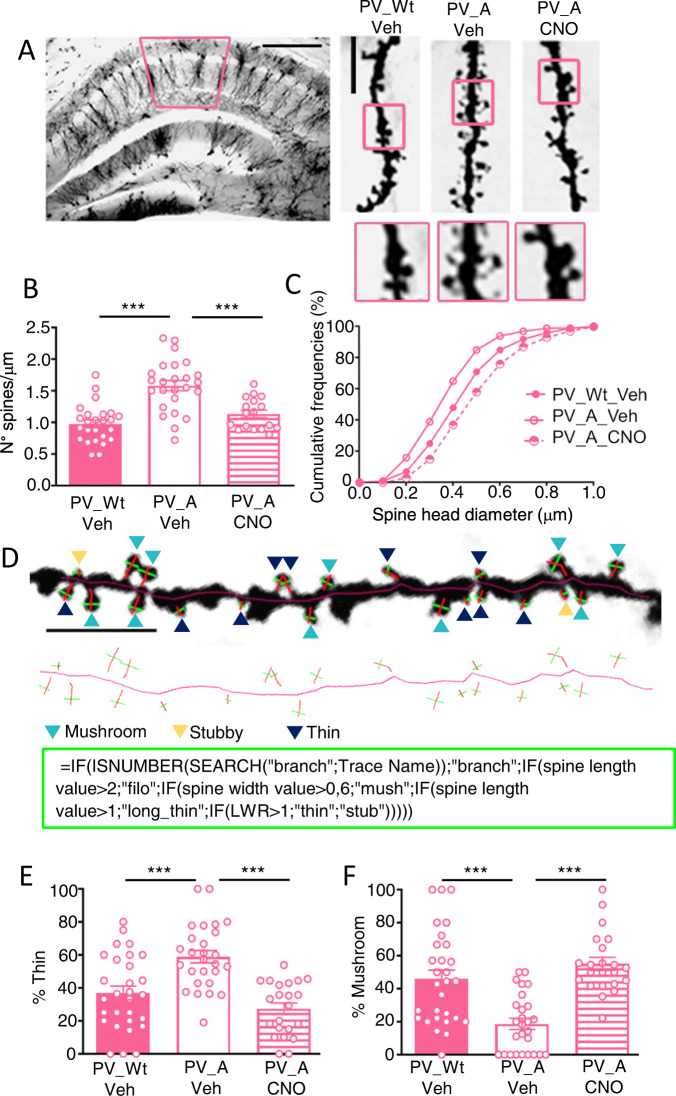
Chemogenetic activation of inhibitory PV-IN in Ambra1^+/−^ females normalizes autistic-like dendritic spines. **A** Representative Golgi-stained dorsal hippocampal section. Left panel: 5x magnification, scale bar: 250 μm. Right panels: 100x magnification, scale bar: 5 μm. **B** Histogram reporting the mean spine density (mean ± s.e.m.): number of spines is similar in PV_Wt/Veh and PV_A/CNO females [PV_Wt/Veh (*N* = 6, 25 neurons), PV_A/Veh (*N* = 5, 25 neurons), PV_A/CNO (*N* = 4, 17 neurons), F_(2,64)_=21.63*, p* = 0.0001; PV_A/Veh vs PV_A/CNO, *p* = 0.0001; PV_Wt/Veh vs PV_A/CNO, *p* = 0.55]. **C** Spine head diameters length are expressed as cumulative frequencies (~1000 spines per group). Spine head diameters in PV_A/CNO females exceed those in PV_Wt/Veh females. (Kolmogorov Smirnoff (KS) test: PV_Wt/Veh vs PV_A/Veh, KS: D = 0.243, p < 0.001; PV_A/Veh vs PV_A/CNO, D = 0.431 *p* < 0.001; PV_Wt/Veh vs PV_A/CNO, D = 0.259, *p* < 0.001). **D** Upper panel: enlarged 100x magnification (scale bar: 5 μm) of a representative dendritic segment where dendritic spines types of CA1 pyramidal neurons are pointed out (mushroom spines by light blue arrows, stubby by yellow arrows and thin by dark blue arrows). Immediately below, the skeleton of the same dendritic segment with the visualization of the neck length and head diameter of each spine. Bottom square: the critical formula used for objective classification of spine-type (see methods for details). **E, F** Histograms reporting the percentage of thin (**E**) and mushroom (**F**) spines: percentage of thin (**E**) and mushroom (**F**) spines is similar in PV_Wt/Veh and PV_A/CNO females [PV_Wt/Veh (*N* = 4, neurons: 8; segments: 29), PV_A/Veh (*N* = 4, neurons: 8; segments: 27), PV_A/CNO (*N* = 4, neurons: 8; segments: 22). Thin spines: F_(2,75)_=16.68, *p* = 0.00002; PV_Wt/Veh vs PV_A/Veh, *p* = 0.0002; PV_A/Veh vs PV_A/CNO, *p* = 0.0001 = ; PV_Wt/Veh vs PV_A/CNO, *p* = 1; Mushroom spines: main effect Kruskal-Wallis H = 28, *p* = 0.0001; Dunn’s multiple comparison PV_Wt/Veh vs PV_A/Veh, p = 0.001; PV_A/Veh vs PV_A/CNO, p = 0.001]. Data are expressed as mean ± s.e.m. *p < 0.05, **p < 0.01, ***p < 0.001.

A neural trait of ASDs patients examined post-mortem, and of ASD mice models, is the increased number of dendritic spines with an immature morphology ubiquitously observed in cortical and subcortical pyramidal neurons [31, 32]. Consistently with the presence of this ASD neural symptom in Ambra1^+/−^ females, we reported that these mice display an increased number of spines with reduced spine-head diameters compared to Wt mice [13]. Whether treatments which rescue ASD-like behaviors also correct dendritic spine defects in ASD mouse models has not yet been investigated. We therefore analyzed the number, the head diameter and the category of spines in Golgi-stained CA1 neurons (Fig. 4A–F) in vehicle-drinking PV_Wt females and vehicle- or CNO solution-drinking PV_A females. In this set of experiments CNO was diluted in the drinking water 24 h before mice were sacrificed for dendritic spine analysis. In line with our previous report [13], vehicle-drinking PV_A females exhibited more spines (Fig. 4B) with reduced spine head diameters (Fig. 4C) compared to vehicle-drinking PV_Wt females. Remarkably, CNO-dilution in the drinking water fully rescued the number of spines in PV_A females that were diminished to the level of vehicle-drinking PV_Wt females. The treatment also increased spine head diameters which, however, were found even larger in CNO-drinking PV_A females compared to vehicle-drinking PV_Wt females. Consistent with these findings, our further analysis of the classification of CA1 dendritic spines (Fig. 4D) revealed in vehicle-drinking PV_A females an increased percentage of thin spines (Fig. 4E) with concurrent reduction in the percentage of mushroom spines (Fig. 4F) compared with vehicle-drinking PV_Wt females. These opposite spine changes were fully rescued in CNO solution-drinking PV_A females even though the percentage of stubby and long-thin spines did not vary among the three groups (Supplementary Figure S2A and B). This observation reveals that the treatment strongly impacts the anatomy of CA1 neuron dendrites as it does not merely rescue the immature morphology of spines but triggers oversized spine heads potentially hosting more robust synapses.

### Chemogenetic activation of PV-IN normalizes hippocampal mRNA ERs in Ambra1^+/−^ females

**Fig. 5:**
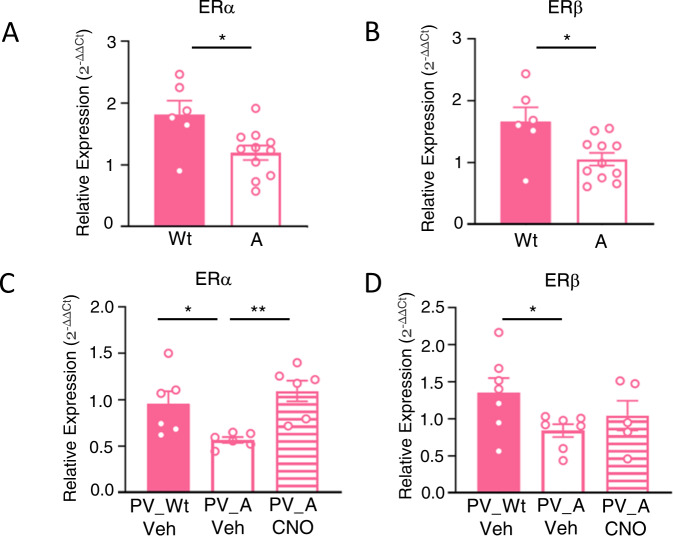
Chemogenetic activation of inhibitory PV-IN in Ambra1^+/−^ females normalizes hippocampal mRNA ERs. **A, B** Histogram reports the relative expression (2-ΔΔCt) of estrogen receptors α (ERs α) and β (ERs β) in the hippocampus of Wt and Ambra1^+/−^ females. Baseline levels of ER α and β are significantly decreased in Ambra1^+/−^ females compared to Wt females. (Wt (*N* = 6), A (*N* = 11), for ERα: t_(15)_=2.76, *p* = 0.0145; for ERβ: t_(15)_=2.746, *p* = 0.015). **C, D** Histogram reports the relative expression (2-ΔΔCt) of estrogen receptors α (ER α) and β (ER β) in the hippocampus of PV_Wt/Veh females and PV_A females injected with Veh or CNO. ER α and β levels are decreased in PV_Ambra1^+/−^ compared to PV_Wt females. **C** ER α deficit is significantly rescued in PV_A/CNO females, whereas no differences in (**D**) ER β level are found in PV_A/CNO females, as compared to their vehicle counterpart. [For ER α: PV_Wt/Veh (*N* = 6), PV_A/Veh (*N* = 6) and PV_A/CNO (*N* = 6); F_(2,15)_=6.96, *p* = 0.007; PV_Wt/Veh vs PV_A/Veh *p* = 0.05, PV_A/Veh vs PV_A/CNO *p* = 0.007. For ER β: PV_Wt/Veh (*N* = 7), PV_A/Veh (*N* = 7) and PV_A/CNO (*N* = 5); F_(2,16)_=2.761, *p* = 0.09; PV_Wt/Veh vs PV_A/Veh: t_(12)_=2.410, *p* = 0.032; PV_A/Veh vs PV_A/CNO: t_(10)_=1.04, *p* = 0.32)]. *p < 0.05, **p < 0.01.

ASD are more frequently observed in males than in females [33, 34] and, consistently with the view that estrogens protect from ASD [35, 36], reduced expression of ERs has been reported in the brain of ASD patients and animal models of both sexes [37, 38]. In the brain, ERs exist in two main forms, ER α and ER β, with different patterns of tissue expression [39]. Although it has been shown that expression of ER β is significantly decreased in the middle frontal gyrus of ASD patients [37], local brain differences in ERs expression remain unexplored in ASD mouse models. Given the causal role of PV-IN loss in the autistic-like phenotype of Ambra1^+/−^ females, and the rescuing effects produced by increasing PV-IN activity, we hypothesized that ERs could be selectively decreased in the hippocampus of autistic females and reinstated at a physiological level by activation of excitatory DREADD in PV-IN. We first compared the baseline expression of hippocampal mRNA ER α and β between Wt and Ambra1^+/−^ females (Fig. 5A and B). In line with our prediction, ER α (Fig. 5A) and β (Fig. 5B) levels were significantly lower in Ambra1^+/−^ females than in Wt females and, consistently with a specific role for decreased ER levels in the autistic phenotype of Ambra1^+/−^ females, no difference for any ER subclass was found between Ambra1^+/−^ males and Wt males which do not express any autistic trait (Supplementary Figure S2C and D). Then, additional groups were used as control for a possible reinstatement of physiological ER levels upon activation of excitatory DREADD in PV-IN. Samples were collected 70 min after CNO injection, a time frame in which fluctuation of mRNA has been reported upon DREADDs stimulation [40, 41]. Results first confirmed the reduction of ER α (Fig. 5C) and β (Fig. 5D) in Veh-PV_A females compared to Veh-PV_Wt females and, importantly, provided the first evidence that revealed CNO injections in PV_A females fully normalized ER α levels (Fig. 5C) but not ER β (Fig. 5D).

## Discussion

Our findings show that a chemogenetic manipulation which augments the PV-IN inhibitory input onto CA1 PN in Ambra1^+/−^ females rescues their autistic-like behavioral and dendritic spines alterations and concurrently normalizes their decreased levels of hippocampal estrogen receptors, the latter observation providing the first evidence that variations in hippocampal neural activity locally modulate expression of these receptors in a model of female autism.

From the seminal study showing that optogenetic manipulations which augment PN excitability in the mPFC of Wt mice were sufficient to enhance gamma waves frequency and to reduce social interactions [42], there is now extensive evidence that opposing optogenetic or chemogenetic manipulations, which enhance PV-IN, or reduce PN, activity in the mPFC of genetic ASD mouse models decrease hyperexcitability and rescue autistic–like behaviors [8–10]. These data definitively emphasize the crucial role of mPFC hyperexcitable neurons in ASD even though neuronal hyperexcitability in motor cortex [6] or cerebellar [7] regions was also found to be associated with the manifestation of autistic symptoms. Paradoxically, although hippocampal CA1 neurons were found hyperexcitable in the valproic acid rat model of ASD [43, 44], there is not yet evidence that mice with mutations in ASD susceptibility genes exhibit hyperexcitability in hippocampal networks. Our findings therefore provide evidence that destabilization of hippocampal circuits associates with the manifestation of ASD-like behaviors, but exclusively in autophagy-deficient females, which otherwise show disruption of hippocampal ER. Interestingly, if the rescue of social and attentional deficits (Fig. 2A–G) obtained by increasing PV-IN inhibitory activity reveals the causal role of the diminished hippocampal PV-IN inhibitory input in this model of female autism, the parallel observation that this manipulation also reinstates physiological level of ER expression (Fig. 5C and D) identifies an autophagy deficiency related mechanism providing a novel and circumscribed target to treat female autism.

### Increasing hippocampal PV-IN activity rescues dendritic spines abnormalities on hippocampal PN in Ambra1^+/−^ females

In spite of the general consensus that ASD-related synaptic dysfunctions largely depend on the ubiquitous presence of abundant and immature dendritic spines in ASD brain circuits [31, 32, 45], whether turning up the inhibitory tone in those circuits stably rectifies, autism-like abundant and immature dendritic spines has not yet been demonstrated. To our knowledge, optogenetic in vivo tools extensively used to rescue autistic-like behaviors in ASD mice did not address the dendritic spines issue because of limitations associated with acute [46] or chronic [47] stimulation. In this regard, chemogenetic appears more appropriate to probe stable variations in neuronal network structure and function since long-lasting/non_invasive CNO delivery (e.g., dilution in drinking water) considerably extends the time the artificial receptor is activated without side effects are detected [48, 49]. By showing that delivering CNO during a 24 h period normalizes spine density and spine head diameters in autistic mice (Fig. 4B–F), our findings provide the first evidence that the ASD phenotype can be rescued at both the systems and circuit level. Of note, and in apparent contrast with our findings, one injection of CNO in the locus coeruleus of ASD mice bearing 16p11.2 deletion [50] shortened the abnormally prolonged process of spine reorganization in the primary motor area during motor training. CNO was, however, delivered during in vivo two-photon Ca2+ imaging of spines and therefore captured rapidly occurring unstable spines changes which differ from the stabilized wiring changes detectable in ex-vivo spine imaging.

### Acute chemogenetic manipulations of excitability in CA1 PN neurons opposite to those carried out in PV-IN neurons rescue ASD-like behavioral traits

Mice with ASD-relevant mutations intrinsically show a reduction in mPFC PV-IN activity which diminishes the inhibitory input onto PN and triggers autistic-like behaviors [51]. The fact that these abnormalities are entirely rescued either by enhancing PV-IN or reducing PN activity means that, at least in the neocortex, these opposing manipulations are functionally equivalent. One peculiarity of PV^+^ neurons is that a majority of those are fast-spiking (FS) cells, which innervate both the perisomatic and proximal dendritic domains of excitatory PN [52]. Although this dense pattern of innervation authorizes to consider them as the principal regulators of synaptic inhibition in any brain region, PV-IN represent about 40% of the GABAergic interneuron population in the mPFC [53] and only 25% in the CA1 subfield of the hippocampus [30]. Considering that other IN-subtypes (e.g., CCK and SST) are similarly expressed in the two regions [54], it was of primary interest examining whether opposing manipulations of hippocampal PV-IN and PN are also functionally equivalent in rescuing the autistic like phenotype of Ambra1^+/−^ females. We found that hippocampal injections of inhibitory DREADDs with a CaMKII promoter aimed at decreasing PN activity entirely replicated the rescue of behavior produced by excitatory DREADDs manipulations of PV-IN activity. This observation therefore reveals that, independently from differences in their regional expression, PV-IN exert the same inhibitory control on PN in hippocampus and mPFC, and that their dysregulation in each of these two regions is equally susceptible to trigger ASD.

### Theoretical and clinical implications

ASD and hippocampus. The central role attributed to mPFC neuronal alterations in ASD derives from observations that major executive functions governed by this region (e.g. attention, response inhibition, cognitive flexibility and working/prospective/social memory) are impaired in autistic patients [55, 56]. Nevertheless, evidence that these patients also show structural and connectivity defects in temporal lobe regions suggests that part of their cognitive alterations depends on impaired hippocampal functions [57]. For example, abnormal hippocampal activation associated with impairments in a reciprocal social interaction task has been ascribed to a deficit in processing spatial and contextual information which prevents socially relevant events to be encoded in spatial and contextual frames [58, 59]. Also, defects in face recognition are associated with alterations in white matter tracts between the hippocampus and the mid-fusiform gyrus [60]. In mice, the selective impairment in social memory produced by lesioning the hippocampal CA2 subfield points to a critical role of this region in encoding and retrieving information relative to previous social experiences [61] among which defective recognition of individuals seems to be primordial [62]. It becomes therefore increasingly evident that the neural substrate of ASD has to be identified at the circuit level where alterations at multiple nodes [63–65] jointly shape the ASD phenotype. Supporting this view, all the autistic traits detected in the present study involve attention and working/recognition memory, that is, cognitive operations which depend on a hippocampal-prefrontal interplay [66]. This, in turn, explains why reducing neuronal excitability in these two regions similarly rescues autistic-like behaviors.

ASD and gender. ASD is more prevalent in males than females [33, 34] with a sex ratio ranging between 2:1 to 3:1. On the one hand, sociocultural influences which lead to gender differences in the expression of autistic traits suggest that female ASD is under-diagnosed [67]. On the other, the “female protective effect (FPE)” theory posits that females are both (i) biologically protected, as they need more copy number of genomic variants than males to become autistic [68], and (ii) cognitively protected, thanks to their propensity to mask cognitive deficit [69]. Female hormones modulate the ASD phenotype regardless of gender since ASD male patients examined *post mortem* show a 35% decrease of mRNA expression of ERβ in the middle frontal gyrus compared to controls [37]. Also, a link between the single nucleotide polymorphisms in ERα/β and ASD severity has been identified [70]. Thus, while the intrinsically elevated levels of ER in healthy females provide a biological explanation for the FPE, any mutation or epigenetic factor which produces ERs insufficiency diminishes the FPE and renders them more prone to show an ASD phenotype. Our data (Fig. 5) support this hypothesis by showing that ER α and β levels are dramatically reduced in Ambra1^+/−^ females compared to Wt females, but do not vary between Ambra1^+/−^ and Wt males (Supplementary Figure S2C and D). Noteworthy, DREADDs manipulations which rescue the ASD-like phenotype restores hippocampal levels of ER α, consistently with a rescue of the FPE.

## Conclusions

Autophagy is an intracellular degradation process that removes unnecessary or dysfunctional material through a lysosome-dependent mechanism under the control of autophagy genes (ATG) [71]. In the brain, early loss of autophagy causes neurodevelopmental disorders which include aberrant neuronal morphology, disruption of neurogenesis, and synaptic malfunctions due to defective pruning and neuronal/glial signaling [72]. The *Ambra1* gene, which regulates autophagosome formation in mammals [73], is one rare example of ATGs whose heterozygous deletion in mice produces a sexual strict dimorphism of autistic traits [74]. Thus, if the autistic-like phenotype selectively observed in Ambra1^+/−^ females establishes that autophagy insufficiency represents a major risk factor for female autism, the demonstration that normalizing hippocampal excitability in this genotype blocks the manifestation of autistic traits at the systems and circuits levels, and concurrently restores physiological levels of ERs, opens novel perspectives for treatments specifically designed for autistic females.

## Supplementary information

Supplementary Figures S1-2

Supplementary figure legends

Supplemental Methods
