# Supplementary material for: Chemogenetic rectification of the inhibitory tone onto hippocampal neurons reverts autistic-like traits and normalizes local expression of estrogen receptors in the Ambra1+/- mouse model of female autism
Source: Transl Psychiatry. 2023 Feb 20;13:63. doi: 10.1038/s41398-023-02357-x (PMC9941573; doi:10.1038/s41398-023-02357-x)

Supplementary Figure 1

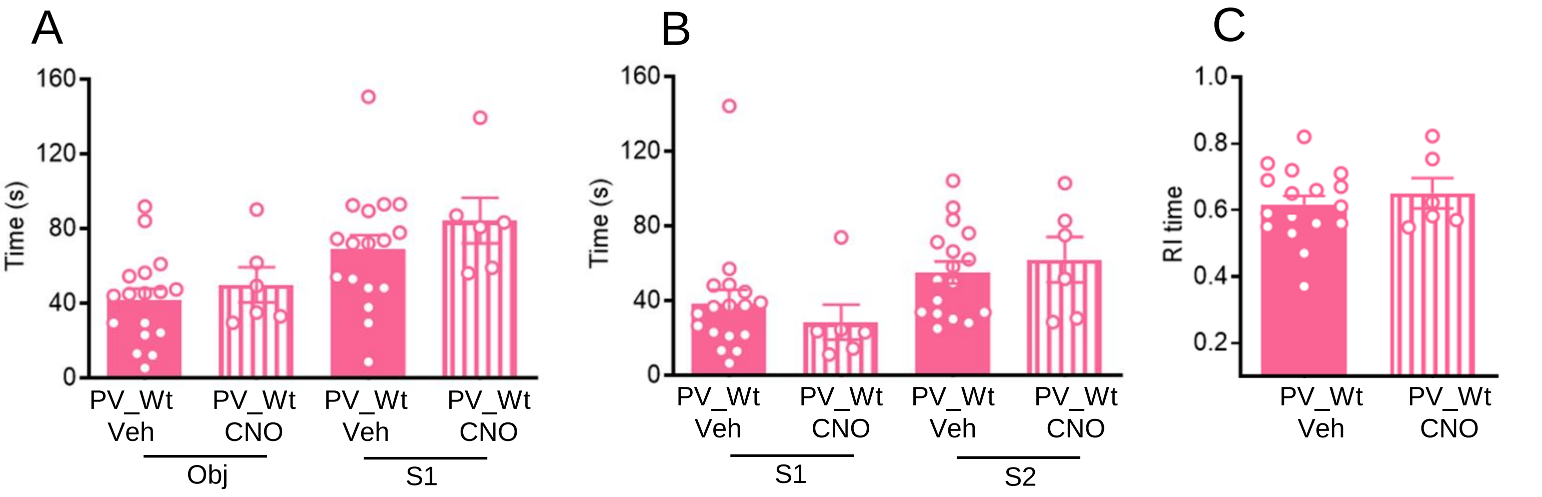


D

| ♀ | PV_Wt/Veh | | PV_Ambra/Veh | | PV_Ambra/CNO | | Stat. | |
|---|---|---|---|---|---|---|---|---|
| | **Mean** | **SEM** | **Mean** | **SEM** | **Mean** | **SEM** | $F_{(2, 41)}$ | **P** |
| **Velocity (cm/s)** | 7.77 | 0.62 | 7.19 | 0.62 | 8.98 | 1.11 | 1.24 | 0.30 |
| **Distance (cm)** | 2415 | 196.6 | 2314 | 256.0 | 2667 | 330.1 | 0.46 | 0.63 |

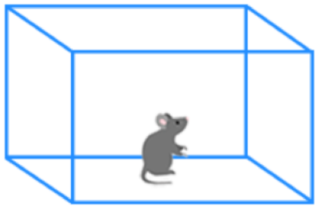

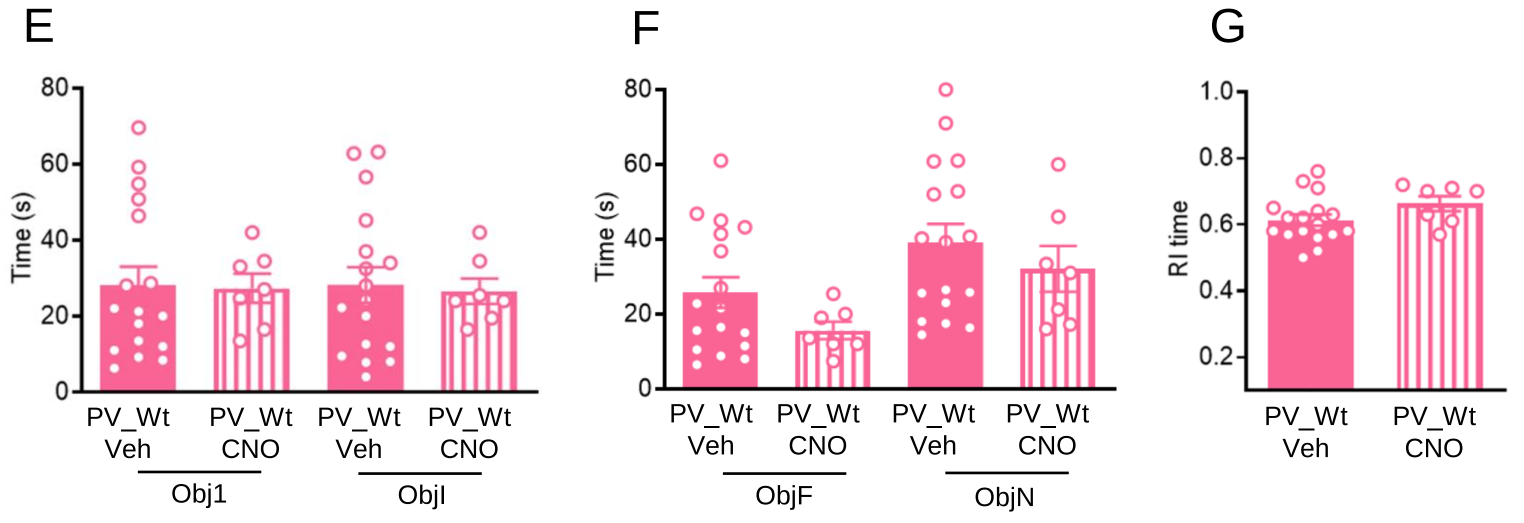


H

| ♀ | Wt/Veh | | Ambra/Veh | | Ambra/CNO | | Stat. | |
|---|---|---|---|---|---|---|---|---|
| | **Mean** | **SEM** | **Mean** | **SEM** | **Mean** | **SEM** | $F_{(2, 20)}$ | **P** |
| **Velocity (cm/s)** | 8.0 | 0.95 | 7.09 | 1.14 | 7.58 | 0.57 | 0.24 | 0.79 |
| **Distance (cm)** | 2533 | 303.3 | 2119 | 340.7 | 2272.1 | 171.1 | 0.53 | 0.60 |

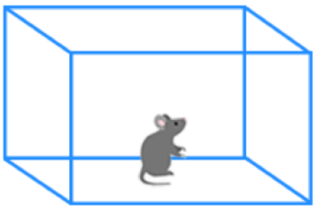

Supplementary Figure 2

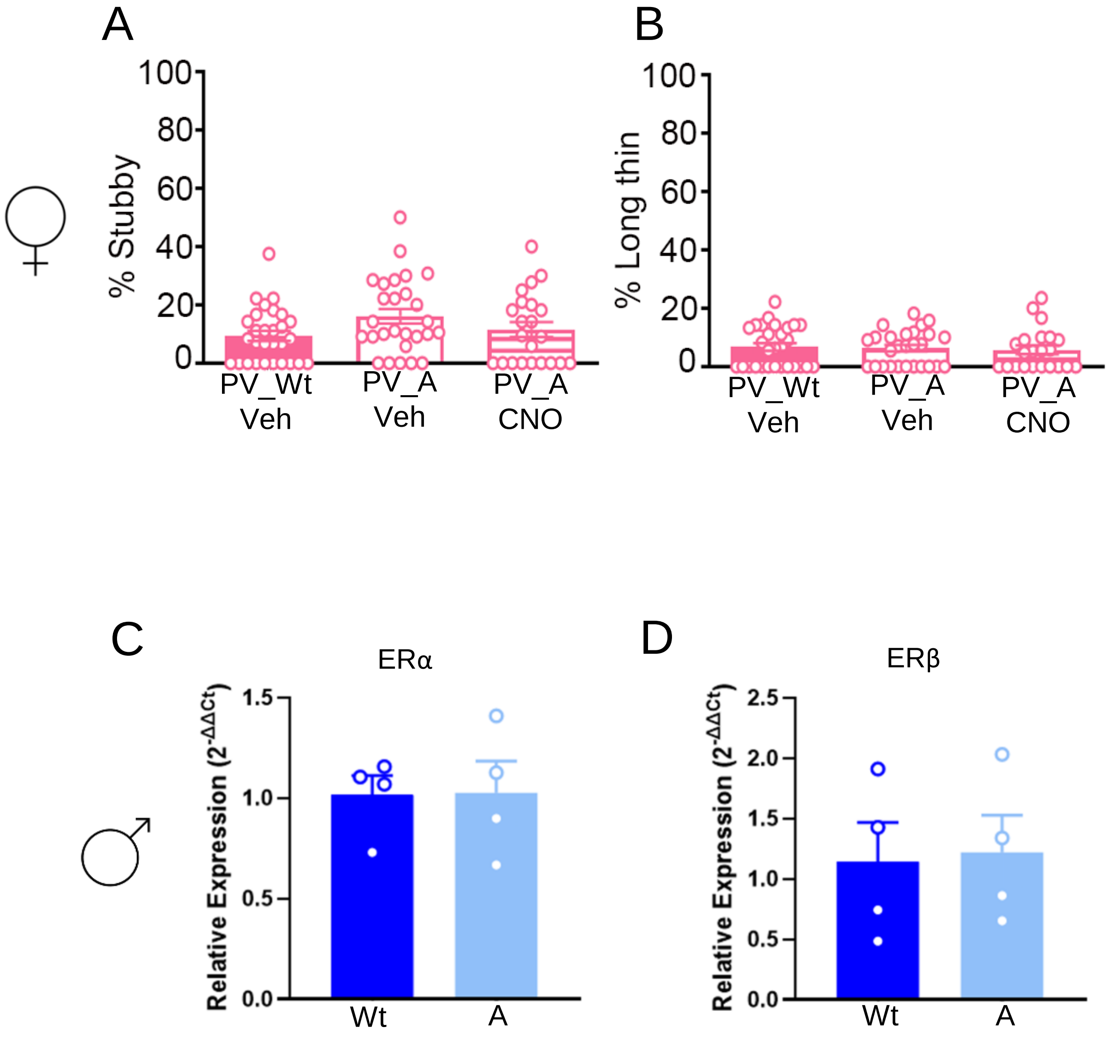

Supplement: Supplementary file 1 — Supplementary Figures S1-2 [file 41398_2023_2357_MOESM1_ESM.pdf]
