# Supplementary material for: Chemogenetic rectification of the inhibitory tone onto hippocampal neurons reverts autistic-like traits and normalizes local expression of estrogen receptors in the Ambra1+/- mouse model of female autism
Source: Transl Psychiatry. 2023 Feb 20;13:63. doi: 10.1038/s41398-023-02357-x (PMC9941573; doi:10.1038/s41398-023-02357-x)
Supplement: Supplementary file 2 — Supplementary figure legends [file 41398_2023_2357_MOESM2_ESM.pdf]

Additional material: supplementary figure legends

S1: A-C Histogram report mean time spent exploring the object (Obj) and the conspecific stranger (S1) during the sociability phase of the TC test in DREADDS-infused PV_Wt/Veh and vehicle-infused PV_Wt/CNO. No differences were detected in exploration of the stranger and of the object among groups (Object exploration: PV_Wt/Veh vs PV_Wt/CNO: $t_{(21)}$=0.70, p=0.48; Stranger exploration PV_Wt/Veh vs PV_Wt/CNO: $t_{(21)}$=1.654, p=0.1). **B** Histogram report mean-time spent exploring the familiar female (S1) and the novel female (S2) during the social novelty phase of the TC test in DREADDS-infused PV_Wt/Veh and vehicle-infused PV_Wt/CNO. No differences were detected in exploration of the familiar female or of the novel female among groups (familiar exploration: PV_Wt/Veh vs PV_Wt/CNO: U=37,5, 0.36; Novel female exploration PV_Wt/Veh vs PV_Wt/CNO: $t_{(21)}$=0.56, p=0.57). **C** Histogram reporting the mean recognition index (RI) calculated during the social novelty phase of TC in DREADDS-infused PV_Wt/Veh and vehicle-infused PV_Wt/CNO. No differences were detected in RI among groups ($t_{(21)}$=0.64, p=0.52. (PV_Wt/Veh (N=17), PV_Wt/CNO (N=6)).

S1: D Table showing the velocity (cm/s) and distance (cm) traveled by Veh-injected PV_Wt and Veh- or CNO-injected PV_A females during the habituation phase of NOR test. No differences are detected among groups. Data are reported as mean ± s.e.m.

S1: E-G. E Histogram report mean time spent exploring the two identical objects (Object 1 (Obj1) and Identical Object (Obj I)) during the object exploration phase of the NOR test in DREADDS-infused PV_Wt/Veh and vehicle-infused PV_Wt/CNO. Data indicate that regardless of treatment all mice similarly explore the identical objects (Obj1: PV_Wt/Veh vs PV_Wt/CNO U=51.5 p= 0.62; ObjI: PV_Wt/Veh vs PV_Wt/CNO $t_{(22)}$=0.21, p=0.83). **F** Histogram report mean time spent exploring the familiar object (ObjF) and the novel object (Obj N) during the test phase of the NOR by DREADDS-infused PV_Wt/Veh and vehicle-infused PV_Wt/CNO. No differences were detected among the two groups in the exploration of the familiar object or in the exploration of the novel object. **(**ObjF: PV_Wt/Veh vs PV_Wt/CNO $t_{(22)}$=1,65, p=0.13; ObjN: PV_Wt/Veh vs PV_Wt/CNO

$t_{(22)}$=0.81 p=0.4). **G** Histogram reporting the mean recognition index (RI) calculated during the test phase of NOR test in DREADDS-infused PV_Wt/Veh and vehicle-infused PV_Wt/CNO. No differences in the RI were detected among groups ($t_{(22)}$=1.65 p= 0.10) (PV_Wt/Veh (N=17), PV_Wt/CNO (N=7)).

S1: H Table showing the velocity (cm/s) and the distance (cm) traveled by Veh-injected Wt and Veh- or CNO-injected A females during the habituation phase of NOR test. No differences are detected among groups. Data are reported as mean ± s.e.m.

S2: A,B Histograms reporting the percentage of stubby **(A)** and long thin **(B)** spines in PV_Wt/Veh, PV_A/Veh and PV_A/CNO female. No differences are detected among the three groups [PV_Wt/Veh (N=4, neurons: 8; segments: 29), PV_A/Veh (N=4, neurons: 8; segments: 27), PV_A/CNO (N=4, neurons: 8; segments: 22). Stubby: Kruskal-Wallis, H=4.2, p=0.12; long thin: H=0.4, p=0.78].

C,D Histograms report relative expression (2-ΔΔCt) of estrogen receptors α (ERs α, Panel **C**) and β (ERs β, Panel **D**) in the hippocampus of A and Wt male mice. No differences in their ER basal levels are shown between the two groups. Wt (N=4), A (N=4); For ERα: WT vs A: $t_{(6)}$=0.056; p=0.95; For ERβ: WT vs A: $t_{(6)}$=0.179; p=0.86. Data are expressed as mean ± s.e.m.
