# Supplementary material for: Chemogenetic rectification of the inhibitory tone onto hippocampal neurons reverts autistic-like traits and normalizes local expression of estrogen receptors in the Ambra1+/- mouse model of female autism
Source: Transl Psychiatry. 2023 Feb 20;13:63. doi: 10.1038/s41398-023-02357-x (PMC9941573; doi:10.1038/s41398-023-02357-x)
Supplement: Supplementary file 3 — Supplemental Methods [file 41398_2023_2357_MOESM3_ESM.pdf]

Additional Material: Supplementary Methods

Animals

Experiments were carried out in accordance with the ethical guidelines of the European Council Directive (2010/63EU) and the Italian Health Ministry (Art.31, D.Lgs 26/2014). Approval was obtained from the Italian Ministry of Health (protocol #357/2018). PV_A and PV_WT littermates were obtained by crossing Ambra1$^{+/-}$ males with homozygous PV-Cre$^{+/+}$ females (JAX stock #017320). Heterozygous Ambra1$^{+/-}$ were obtained by breeding Ambra1$^{+/-}$ males with C57BL/6J females. Animals were housed in a temperature- and humidity-controlled environment (free access to food and water; 12 h dark/light cycle).

Genotyping

To determine genotype, DNA was isolated from tails digesting the tissues at 56 °C in lysis buffer containing (in mM): 50 Tris-HCl pH 8.0, 100 EDTA pH 8.0, 100 NaCl, 1% SDS, and 1 µg/µl proteinase K (NZYTech). For genotyping, Ambra1 primer sequences are: 5'-TCCCGAAAACCAAAGAAGAAGA-3'; 5'-CCCAGTCACGACGTTGTAAAA-3'. DNA amplification and PCR products were analysed as in Nobili et al. [1]; PV-Cre was confirmed as indicated by Jackson Laboratory.

DREADDs injections and activation

Surgery Mice at PND45 were anesthetized with a mixture of Rompun (20 mg/ml, 0.5 ml/kg; Bayer) and Zoletil (100 mg/ml, 0.5 ml/kg; Virbac) and fixed on a stereotaxic apparatus (David Kopf, USA). Following a longitudinal-medial incision of the scalp, two holes were drilled in the skull in correspondence to the CA1 (AP: −2.2; ML: ±2 mm from bregma [2]). A cannula (0.1 mm diameter) connected to a Hamilton micro-syringe attached to an infusion pump

(Harvard Apparatus, PHD, 2000) was descended dorsoventrally in each hemisphere (−1.7 mm from the bregma) to infuse bilaterally at a low rate (0.3 μL/min) 0.7 μL of the viral vector. After completion of the injection, the injector tip was kept in place for 5 minutes to prevent solution backflow and then carefully retracted to prevent leakage. After suturing, mice were re-housed in their home-cages for 4 weeks (time required to allow the expression of the viral construct) and monitored during recovery.

Viral vectors We used:

- AAV-hSyn-DIO-hM3D(Gq)-mCherry (#44361; Addgene) in PV_A females to enhance CA1 PV-IN activity;
- AAV5/CaMKIIa-hM4D(Gi)-mCherry (AV6334; University of North Carolina Vector Core) in Ambra1$^{+/-}$ females to inhibit CA1 PN activity.

Clozapine-N-oxide (CNO Sigma) was dissolved in dimethyl sulfoxide (DMSO) to obtain a 10 mg/ml CNO solution. *For i.p. injections*, CNO was then mixed with 0.9% saline, and injected at a dose of 5 mg/kg. The Vehicle (Veh) group received only DMSO dissolved in 0.9% saline. For CNO administration in drinking water, preliminary experiments were first carried out to determine the amount of water a mouse consumes per day (approximately 3–5 ml of water/day). Based on the number of mice per cage, the amount of water required for 1 day was calculated for each cage, and 5 mg/kg of CNO/mouse/day was added to the water. We added sucrose (1%) to the drinking water to encourage CNO consumption. The control group received vehicle (wather+DMSO) with 1% sucrose.

DREADDs specificity was confirmed by co-labelling of mCherry and PV or αCamKII. Mice were anesthetized with Rompun (20 mg/ml, 0.5 ml/kg, i.p., Bayer) and Zoletil (100 mg/ml, 0.5 ml/kg, Virbac) and perfused transcardially with 50 ml saline followed by 50 ml 4%

paraformaldehyde in Phosphate Buffer (PB; 0.1 M, pH 7.4). Brains were removed and post-fixed in paraformaldehyde (4°C) and then immersed in 30% sucrose solution (4°C) until sinking. Brains were cut into 30 µm-thick coronal sections with a cryostat and slices were collected in PB. Selected sections were processed with primary antibody in PB containing Triton 0.3% over night. After three washes in PB, sections were incubated with secondary antibodies and NeuroTrace. Primary antibody: PV (1:500; Sigma-Aldrich; P 3088; RRID: AB_477329); αCamKII (#13-7300; Thermo Fisher; RRID:AB_2533032). Secondary antibodies: Alexa Fluor 488 donkey anti-mouse IgG (1:200; Thermo Fisher Scientific; #R37114; RRID: AB_2556542), NeuroTrace 435/455 (1:200; Thermo Fisher Scientific; #N21479). Sections were coverslipped with Aqueous Mounting Media (Sigma-Aldrich) and examined under a confocal laser-scanning microscope (LSM700, Zeiss). The immunofluorescence labelling specificity was confirmed by the omission of primary antibodies and the use of normal serum instead (negative controls).

Electrophysiology

Brain slicing Slicing was performed as previously described [1]. Parasagittal slices containing the dorsal hippocampus (280 µm) were obtained with a Leica VT1200S vibratome in chilled bubbled (95% O_2, 5% O_2) ice-cold sucrose-based solution (in mM): KCl 3, NaH2PO4 1.25, NaHCO3 26, MgSO4 10, CaCl2 0.5, glucose 25, sucrose 185; ~300 mOsm, pH 7.4), incubated in artificial cerebrospinal fluid (aCSF; containing in mM): 124 NaCl, 3 KCl, 1.25 NaH2PO4, 26 NaHCO3, 1 MgSO4, 2 CaCl2, 10 glucose, ~300 mOsm, pH 7.4) at 34°C for 40 min, and transferred at room temperature for at least 30 min before recordings.

Patch clamp recordings A single brain slice was transferred to a recording chamber of an upright microscope (Zeiss Axioskop 2-FS; Germany) and continuously perfused (3 mL/sec, 32°C) with aCSF. Whole-cell recordings were made from CA1 pyramidal neurons, identified

using a 60x magnification. Recordings were performed with Axon 700B amplifier using 4 kHz low pass-filter, digitized at 20 kHz with Digidata 1400A and computer-saved using Clampex 10.3 (Molecular Devices, Sunnyvale, CA). No liquid junction potential correction was applied. Recording electrodes (3-4.5 MΩ) were pulled from thin-wall borosilicate glass tubes and filled with (in mM): 140 CsCl, 1 MgCl2, 10 HEPES, 2.5 QX314-Cl, 4 Mg-ATP (~290 mOsm, pH 7.3). For inhibitory currents the extracellular aCSF contained (in μM) 10 NBQX (Abcam), 50 D-AP5 (Abcam), 1 CGP55845 (Sigma-Aldrich) and 5 CNO to block AMPA/kainate, NMDA and $GABA_B$ receptors. For sIPSC recordings from PV_A females (-70mV), mice were injected intraperitoneally with either vehicle or CNO 40 minutes before slicing, and slices were kept in aCSF containing 0.01 mM CNO. For analysis, a one-minute-long analysis window was scanned for the detection of inhibitory events; single currents were detected manually with the threshold crossing method of Clampfit 10.3 (Molecular Devices, CA) and analysed for amplitude and instantaneous frequency; parameters were tested for time stability using Spearman's rank-order correlation test; segments of events that showed instability during the experiment were excluded. At least 400 events were analyzed for each experiment.

Dendritic Spine analysis

Golgi Cox Staining Mice were deeply anaesthetised with a mixture of Rompun (20 mg/ml, 0.5 ml/kg; Bayer) and Zoletil (100 mg/ml, 0.5 ml/kg; Virbac) and perfused transcardially with 0.9% saline. Brains were dissected and impregnated in a Golgi-Cox solution (1% potassium dichromate, 1% mercuric chloride and 0.8% potassium chromate) at room temperature for 6 days. On the 7th day, brains were transferred in 30% sucrose solution and sectioned with a vibratome. Coronal sections (100 μm) were collected and stained according to the method described by Gibb and Kolb [3].

Spine Density and morphology Spine analysis was performed on dendritic segments of apical dendrites of CA1 pyramidal neurons. Neurons were first identified with a light microscope (Leica DMLB) under low magnification (20x/NA 0.5). Subsequently, quantification of dendritic spines was done online under ×100 magnification using a Qimaging Qicam Fast1394 camera connected to the microscope. From each brain, three-five neurons were selected and, on each neuron, five 30–100 μm dendritic segments were randomly selected for spine counts. Only protrusions with a clear connection of the spine head to the shaft of the dendrite were counted as spines using the Neurolucida software. Statistical comparisons were made on single neuron values obtained by averaging the number of spines counted on segments of the same neuron. Furthermore, spine head diameters were measured on previously acquired images (Motic Live Imaging software) by measuring spine head diameter parallel to the dendrite using the ImageJ (NIH, USA) software. Spine head diameter values were expressed as cumulative frequencies.

Supplemental References

1. Nobili A, Krashia P, Cordella A, La Barbera L, Dell'Acqua MC, Caruso A *et al.* Ambra1 shapes hippocampal inhibition/excitation balance: role in Neurodevelopmental Disorders. *Mol Neurobiol* 2018; 55: 7921-7940.

2. Franklin KBJ, Paxinos G, 1997. The Mouse Brain in Stereotaxic Coordinates Academic Press, San Diego. CA, USA.

3. Gibb R, Kolb B. A method for vibratome sectioning of Golgi-Cox stained whole rat brain. *J Neurosci Methods* 1998; 79: 1-4.
